# Antibiotic resistance trends of ESKAPE pathogens in Kwazulu-Natal, South Africa: A five-year retrospective analysis

**DOI:** 10.4102/ajlm.v7i2.887

**Published:** 2018-12-06

**Authors:** Yogandree Ramsamy, Sabiha Y. Essack, Benn Sartorius, Miriam Patel, Koleka P. Mlisana

**Affiliations:** 1 Department of Medical Microbiology, School of Laboratory Medicine and Medical Sciences, Antimicrobial Research Unit, University of KwaZulu-Natal, National Health Laboratory Services, Durban, South Africa; 2 Antimicrobial Research Unit, College of Health Sciences, University of KwaZulu-Natal, Durban, South Africa; 3 School of Nursing and Public Health, University of KwaZulu-Natal, Durban, South Africa; 4 National Health Laboratory Services, University of KwaZulu-Natal, Durban, South Africa

## Abstract

**Background:**

To combat antimicrobial resistance, the World Health Organization developed a global priority pathogen list of antibiotic-resistant bacteria for prioritisation of research and development of new, effective antibiotics.

**Objective:**

This study describes a five-year resistance trend analysis of the ESKAPE pathogens: *Enterococcus faecium, Staphylococcus aureus, Klebsiella pneumoniae, Acinetobacter baumannii, Pseudomonas aeruginosa and Enterobacter* spp., from Kwazulu-Natal, South Africa.

**Methods:**

This retrospective study used National Health Laboratory Services data on 64 502 ESKAPE organisms isolated between 2011 and 2015. Susceptibility trends were ascertained from minimum inhibitory concentrations and interpreted using Clinical and Laboratory Standards Institute guidelines.

**Results:**

*S. aureus* was most frequently isolated (*n* = 24, 495, 38%), followed by *K. pneumoniae* (*n* = 14, 282, 22%). Decreasing rates of methicillin-resistant *S. aureus* (28% to 18%, *p* < 0.001) and increasing rates of extended spectrum beta-lactamase producing *K. pneumoniae* (54% to 65% *p* < 0.001) were observed. Carbapenem resistance among *K. pneumoniae* and *Enterobacter* spp. was less than 6% during 2011–2014, but increased from 4% in 2014 to 16% in 2015 (*p* < 0.001) among *K. pneumoniae. P. aeruginosa* increased (*p* = 0.002), but resistance to anti-pseudomonal antimicrobials decreased from 2013 to 2015. High rates of multi-drug resistance were observed in *A. baumanni* (> 70%).

**Conclusion:**

This study describes the magnitude of antimicrobial resistance in KwaZulu-Natal and provides a South African perspective on antimicrobial resistance in the global priority pathogen list, signalling the need for initiation or enhancement of antimicrobial stewardship and infection control measures locally.

## Introduction

The burden of multi-drug resistant (MDR) bacteria has increased substantially worldwide^1^ and infections caused by these organisms continue to pose a great challenge to public health systems and populations at large. MDR is defined as non-susceptibility to at least one agent in three or more antimicrobial categories.^2^ MDR bacterial infections are among the top three threats to global public health. Due to the lack of surveillance programmes, the true extent of antimicrobial resistance in the African region is unknown or underestimated. Additionally there is no formal framework for collaboration among surveillance programmes in the region.^1^ A request was made to the World Health Organization (WHO) by member states in 2017 to develop a global priority pathogen list (PPL) of antibiotic-resistant bacteria to help in prioritising the research and development of new and effective antibiotic treatments.^3^ Due to the high prevalence of multi-drug resistance among ESKAPE bacteria, defined by the Infectious Diseases Society of America as *Enterococcus faecium, Staphylococcus aureus, Klebsiella pneumoniae, Acinetobacter baumannii, Pseudomonas aeruginosa* and *Enterobacter* spp., these pathogens feature prominently in the global PPL of antibiotic-resistant bacteria and are the focus of this article. Members of the ESKAPE group of bacteria ‘escape’ the biocidal action of antibiotics and present new paradigms in pathogenesis, transmission and resistance of infectious diseases.^4^ ESKAPE pathogens with multiple drug resistance mechanisms have been implicated in life-threatening nosocomial infections, especially among critically ill individuals,^5^ presenting clinicians with serious therapeutic challenges.^5^ The global PPL stratifies bacterial pathogens into three priority tiers: critical, high and medium.^3^ Carbapenem-resistant *A. baumannii, P. aeruginosa* and Enterobacteriaceae spp., which includes *K. pneumoniae*, feature in the critical priority tier. Methicillin-resistant, vancomycin intermediate and resistant *S. aureus*, in addition to *E. faecium*, are featured in the high priority tier. The ESKAPE group of bacteria deserve special attention due to their association with antibiotic resistance,^6^ as infections caused by this particular group of pathogens result in high mortality and morbidity rates, increased healthcare costs, diagnostic dilemma and difficulty in the initiation of empirical treatment.^7^ As a control measure to decrease the incidence of infections due to ESKAPE pathogens, site-by-site surveillance studies and antibiograms are necessary to inform effective empiric therapy.^8^ This study assessed trends in annual resistance rates for all ESKAPE pathogens processed over a five-year period from 2011 through to 2015 in KwaZulu-Natal, South Africa.

### Evidence before this study

Pathogen surveillance and antimicrobial resistance surveillance informs effective antimicrobial prescribing, in addition to controlling the spread of resistant pathogens within the local environment. It is well known that dedicated surveillance systems form an integral strategy to combat antibiotic resistance; however, surveillance of antibiotic-resistant bacteria is severely lacking in most African countries. The largest antimicrobial resistance surveillance data set from sub-Saharan Africa was published in 2017 and reflected antimicrobial resistance trends from isolates implicated in bloodstream infections from adults and children in Malawi over a 19-year period were described.^9^ There have been no long-term surveillance data reports describing ESKAPE pathogens and their antimicrobial resistance patterns from KwaZulu-Natal, South Africa, except for national sentinel site surveillance describing antimicrobial resistance among *K. pneumoniae* isolates from bloodstream infections from 2009–2012. Hence, this study aimed at providing a description of ESKAPE pathogens and their antibiotic resistance trends over a five-year period in the province of KwaZulu-Natal, South Africa.

### Added value of this study

The ESKAPE group of bacteria are among the critical and high priority pathogens on the global PPL. This study provides the largest data set of the ESKAPE pathogens gathered from adults and children who presented to the largest public service hospitals in KwaZulu-Natal, South Africa, over a five-year period. The surveillance data provided in this manuscript provide a good baseline surveillance intervention to combat antimicrobial resistance.

### Implications of all the available evidence in this study

The WHO global PPL identifies priority pathogens for the research and development of new antibiotics. Knowledge of these pathogens and their resistance patterns in local contexts is pivotal in our fight against antimicrobial resistance. Despite a decreasing trend in methicillin-resistant *S. aureus* (MRSA), there is emergence and rapid expansion of extended spectrum beta-lactamase (ESBL)-producing *K. pneumoniae* coupled with fluoroquinolone resistance observed in this study. Additionally, increasing resistance to third-generation cephalosporins and amoxicillin-clavulanate is a growing concern, as there is now a greater reliance on the carbapenems to treat infections caused by *K. pneumonia*e. Increasing resistance to carbapenems observed in *K. pneumoniae* is slowly becoming a reality in KwaZulu-Natal and may spell the beginning of the end for carbapenems against Enterobacteriaceae such as *K. pneumoniae.* Continued surveillance will provide valuable information on antimicrobial resistance patterns for ESKAPE pathogens, which will assist in informing empiric antimicrobial therapy.

## Methods

### Ethical considerations

This study was approved by the Biomedical Research Ethics Committee of the University of KwaZulu-Natal (study approval number: BE085/12).

### Study sites

Data collected from August 2011 to December 2015 at nine participating public sector hospitals from two districts across the province of KwaZulu-Natal were utilised in this study. The nine participating hospitals are the largest in the province and represent all levels of healthcare as described in Tables 1 and 2.^10^

**TABLE 1:** Public hospitals and bed numbers in KwaZulu-Natal, South Africa.

Hospital	Level of healthcare	Number of beds
Prince Mshiyeni Memorial Hospital	Level 2 Regional	1200
Edendale Hospital	Level 2 Regional	874
Inkosi Albert Luthuli Central Hospital	Level 4 Central	846
King Edward VIII Hospital	Level 3 Tertiary	799
Addington Hospital	Level 2 Regional	571
R K Khan Hospital	Level 2 Regional	543
Greys Hospital	Level 3 Tertiary	530
Northdale Hospital	Level 1 District	385
Mahathma Gandhi Memorial Hospital	Level 2 Regional	355

*Source*: Health K-NDo. Provincial Hospitals: KwaZulu-Natal Department of Health. 2014 [cited 2018 Jan 15]. Available from: http://www.kznhealth.gov.za/hospitals.htm^10^

**TABLE 2:** Levels of healthcare in KwaZulu-Natal, South Africa.

Level	Healthcare facility
1	**Primary healthcare clinic:** Primary healthcare clinics are the first point in the provision of healthcare. Services such as immunisation, family planning, antenatal care, treatment of tuberculosis, HIV/AIDS counselling, and treatment for common conditions among others are offered here.^10^
	**Community healthcare centre:** Community healthcare centres offer similar services to a primary healthcare clinic with the addition of a 24 h maternity service, emergency care and casualty and a short stay ward.^10^
	**District hospital:** These hospitals receive referrals from and provide generalist support to community health centres and clinics. Diagnostic, clinical and counselling services are provided. Clinical services provided include: casualty, internal medicine, paediatrics, surgery, obstetrics and gynaecology, out-patients, mental health, geriatrics and clinical forensic medical services.^10^
2	**Regional hospital:** These are the second level of healthcare. Regional hospitals receive referrals from and provide specialist support to a number of district hospitals.^10^
3	**Provincial tertiary hospital:** These hospitals receive referrals from and provide sub-specialist support to regional hospitals and are the third level of healthcare. Provincial tertiary hospitals are staffed by specialists and generalists and offer services such as neurosurgery, neurology, plastic and reconstructive surgery, cardiology, urology, paediatric surgery, maxillio-facial surgery, psychiatry, occupational health and orthopaedics.^10^
4	**Central hospitals:** These are the fourth and highest level of healthcare. Central hospitals consist of highly specialised units which together provide an environment for multi-speciality clinical services, research and innovation.^10^

*Source*: Health K-NDo. Provincial Hospitals: KwaZulu-Natal Department of Health. 2014 [cited 2018 Jan 15]. Available from: http://www.kznhealth.gov.za/hospitals.htm^10^

### Bacterial samples

Each participating centre extracted data of all isolates categorised as ESKAPE pathogens. All body sites were considered acceptable sources. Samples submitted for microbiological analysis included blood, urine, catheters (central venous catheter + hemodialysis) and respiratory specimens, in addition to samples from other body sites. Not every patient who presented to a healthcare facility with an infection submitted a sample for microbiological analysis. Additionally, the data presented included organisms from all specimen types; hence, colonisation was not distinguished from infection.

### Antimicrobial susceptibility testing

All participating laboratories in this study subscribed to the National Health Laboratories Services (NHLS) Proficiency Testing Scheme. Evaluations are carried out quarterly and samples for the scheme are prepared with assistance from the National Institute for Communicable Diseases – Centre for Opportunistic, Tropical and Hospital Infections, a division of the NHLS. Thus, all laboratories participating in this study were proficient in testing bacterial samples. All participating laboratories were responsible for sample processing, including isolate identification and susceptibility testing. Pathogen identification was determined using the Vitek 2 (bioMerieux, Marcy l’Étoile, France) platform. Antimicrobial susceptibility testing and minimum inhibitory concentration determinations were performed using the Vitek 2 (bioMerieux, Marcy l’Étoile, France) platform. The results were interpreted according to the criteria of the Clinical Laboratory Standards Institute.^11^ The antimicrobial test panel included: penicillin, ampicillin, amoxicillin-clavulanate, ceftriaxone, cefepime, cefuroxime, cefoxitin, ceftazadime, imipenem, meropenem, ertapenem, piperacillin-tazobactam, amikacin, gentamicin, erythromycin, clindamycin, linezolid, teicoplanin, vancomycin, fusidic acid, mupirocin, tetracycline, oxacillin, rifampicin, nitrofurantoin, colistin, trimethoprim/sulfamethoxazole, ciprofloxacin and tigecycline. Methicillin resistance in *S aureus* was inferred by oxacillin resistance.^11^ The Vitek 2 AST-N255 card was used to perform antimicrobial susceptibility testing on Gram-negative, whereas the AST-P603 card was used for Gram-positive organisms. Presumptive ESBL production in *K. pneumoniae* was determined using the minimum inhibitory concentration for ceftriaxone and ceftazidime as per the Clinical Laboratory Standards Institute guidelines.^11^ Isolates were characterised as susceptible or resistant using Clinical Laboratory Standards Institute-approved breakpoints.^11^

### Data extraction and analysis

All information regarding specimen collection, sampling and patient laboratory results is deposited on the NHLS laboratory database. The use of this database and access to patient information is restricted to laboratory staff working within the NHLS. Thus, data collection was done at the NHLS and entailed extraction of isolate information from this computerised laboratory database. The data received were de-duplicated, anonymised and patient confidentiality was maintained at all times. Isolate information, specimen type and results of antimicrobial susceptibility testing, including minimum inhibitory concentration data, were also extracted. Once the data were extracted, trends in the total number of ESKAPE pathogens and their antimicrobial resistance patterns were determined. These trends were compared over a five-year period.

### Statistical analysis

All data processing and analyses were performed using Stata 13.0 software SE (StataCorp. LP, 2013, College Station, Texas, United States). Categorical data were presented using stratified frequency tables (*n* and %). Trends or associations were assessed using the standard Pearson’s chi-square (χ2) test. If expected cell count in the cross tabulation contained fewer than five observations (sparse numbers), then the Fisher’s exact test was utilised instead. A *p*-value of less than 0.05 was considered statistically significant.

## Results

### Distribution of species by clinical specimens

**FIGURE 1:**
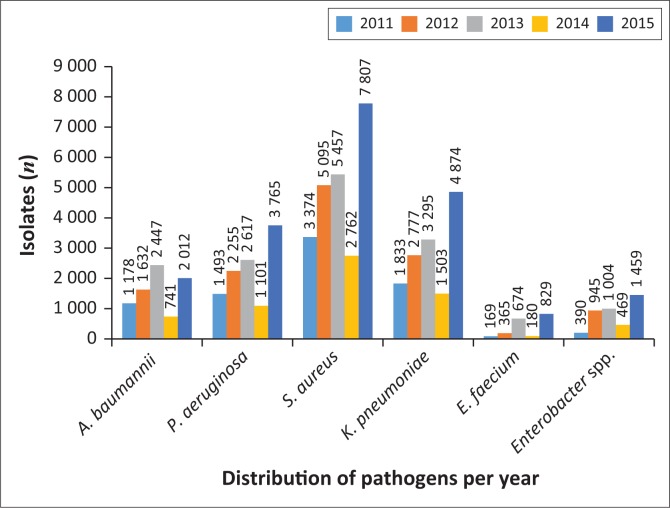
Trends in the number of ESKAPE pathogens isolated from clinical samples collected in public sector healthcare facilities in KwaZulu-Natal from 2011 to 2015.

For the period 2011–2015, a total of 64 502 ESKAPE clinical isolates were recovered from clinical specimens (Figure 1). The distribution of the isolated ESKAPE organisms varied during this period. The clinical specimens included respiratory specimens, blood, urine, catheters, which included central venous and hemodialysis catheters, as well as samples from other sites, including wounds. Respiratory samples included sputum, bronchi alveolar lavage and endotracheal aspirates. Overall, *S. aureus* (24 495, 38.0%) was the most frequently isolated pathogen followed by *K. pneumoniae* (14 282, 22.2%), *P. aeruginosa* (11 231, 17.4%), *A. baumannii* (8010, 12.4%), *Enterobacter* spp. (4267, 6.6%), *E. faecium* (2217, 3.4%) (Table 3 and Figure 1). Similarly, *S. aureus* (3787, 35.8%) and *K. pneumoniae* (3059, 28.9%) were the most frequently isolated species from blood cultures. Gram-negative organisms namely *K. pneumoniae* (2469, 29.2%), *A. baumannii* (2036, 24.1%) and *P. aeruginosa* (2015, 23.8%) were the most common isolates from respiratory samples, followed by Gram-positive species, of which *S. aureus* was the most frequently isolated (1353, 16%). *K. pneumoniae,* a member of the Enterobacteriaceae group, was the predominant species isolated from urine (4997, 55.5%). Catheters included a combination of central venous, arterial and hemodialysis catheters. *A. baumannii* (31.5%), *S. aureus* (23.4%) and *K. pneumoniae* (23.1%) were the most common isolates cultured. Other sample types included wound swabs, pus swabs and aspirates. The most frequently isolated species from other sample types was *S. aureus* (18 397, 53.4%).

**TABLE 3:** Distribution of ESKAPE pathogens isolated from clinical samples collected in public sector healthcare facilities in KwaZulu-Natal from 2011–2015.

ESKAPE pathogen	Blood		Respiratory		Urine		Catheter		Other†		Total	
	*n*	%	*n*	%	*n*	%	*n*	%	*n*	%	*n*	%
***A. baumannii***	1572	14.9	2036	24.1	894	9.9	637	31.5	2871	8.3	8010	12.4
***P. aeruginosa***	596	5.6	2015	23.8	910	10.1	224	11.1	7486	21.7	11 231	17.4
***S. aureus***	3787	35.8	1353	16	484	5.4	474	23.4	18 397	53.4	24 495	38.0
***K. pneumoniae***	3059	28.9	2469	29.2	4997	55.5	468	23.1	3289	9.5	14 282	22.2
***E. faecium***	877	8.3	29	0.3	756	8.4	52	2.6	503	1.5	2217	3.4
***Enterobacter* spp.**	678	6.4	558	6.6	955	10.06	170	8.4	1906	5.5	4267	6.6
**Total**	**10 569**	**100**	**8460**	**100**	**8996**	**100**	**2025**	**100**	**34 452**	**100**	**64 502**	**100**

† , Includes pus swabs, pus and aspirates.

### Antibiotic resistance

During the five-year period, high levels of antibiotic resistance were detected among the ESKAPE pathogens. Overall, the most common resistance pattern in Gram-negative ESKAPE pathogens was resistance to amoxicillin-clavulanate and ceftriaxone. The majority of the *A. baumannii* isolates were resistant to multiple drugs, with over 70% of isolates resistant to all tested antimicrobial agents except colistin, amikacin and gentamicin. Isolates of *P. aeruginosa* remained fairly susceptible to agents like amikacin, piperacillin-tazobactam, meropenem imipenem and colistin. Overall, colistin resistance was less than 10% over the five-year period for the Gram-negative ESKAPE pathogens.

**FIGURE 2:**
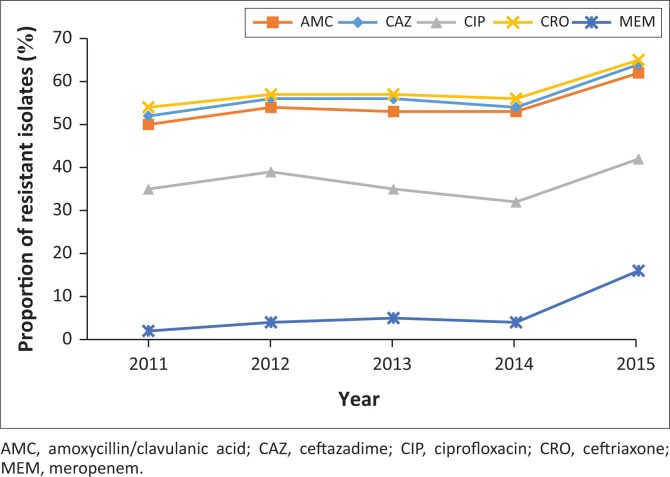
Resistance profile of *K. pneumoniae* (2011–2015).

Between 2011 and 2015, 7788 (54.5%) *K. pneumoniae* isolates were resistant to amoxicillin-clavulanate. More than 50% of all isolates were resistant to third-generation cephalosporins, including ceftriaxone (8326, 58.3%) and ceftazadime (7412, 51.9%), indicative of ESBL production; 5317 (37.2%) isolates were resistant to ciprofloxacin. Of the 14 282 *K. pneumoniae* isolates, 922 (6.4%) were resistant to meropenem (Table 4). Increasing antimicrobial resistance trends were observed in *K. pneumoniae* (Figure 2). Resistance to ceftriaxone increased from 54.6% in 2011 to 65.5% in 2015 (*p* = 0.018). For the period 2011–2014 average resistance to ciprofloxacin was 35%, which increased to 42% in 2015 (*p* = 0.015). Resistance to amoxicillin-clavulanate increased from 50.6% in 2011 and to 62.7% in 2015 (*p* = 0.009). Between 2011 and 2014, resistance to meropenem was 5% or less. This was followed by a significant increase to 16% in 2015 (*p* < 0.001). Colistin resistance in *K. pneumoniae* was approximately 2%, but this was not confirmed by reference or molecular methods. Both European Committee on Antimicrobial Susceptibility Testing and the Clinical Laboratory Standards Institute recommend broth microdilution for antimicrobial susceptibility testing of colistin, but broth microdilution is rarely used in routine microbiology laboratories.^12^ Broth microdilution is not routinely performed at these KwaZulu-Natal public sector microbiology laboratories and as a result they may have failed to detect heteroresistance.

**TABLE 4:** Resistance profiles of Gram-negative ESKAPE pathogens (2011–2015).

Antimicrobial agent	*Acinetobacter baumanni* (8010) % resistant	*Enterobacter spp.* (4267) % resistant	*Klebsiella pneumoniae* (14 282) % resistant	*Pseudomonas aeruginosa* (11 231) % resistant
**AMC**	Not tested	Not tested	54.50	Not tested
**AMK**	18	0.75	4.00	6.30
**CAZ**	77	27.00	51.90	14.20
**CIP**	71	12.00	37.20	13.40
**CRO**	Not tested	Not tested	58.30	Not tested
**CST**	4.6	2.00	2.00	7.50
**GEN**	68	17.50	48.00	17.00
**IMP**	72	3.00	4.00	13.00
**MEM**	73	2.00	6.40	9.70
**TZP**	81	22.50	42.00	15.20

AMC, amoxycillin/clavulanic acid; AMK, amikacin; CAZ, ceftazadime; CIP, ciprofloxacin; CRO, ceftriaxone; CST, colistin; GEN, gentamicin; IMP, imipenem; MEM, meropenem; TZP, piperacillin/tazobactam.

High MDR rates were observed for *A. baumannii* with over 70% of isolates resistant to meropenem, imipenem, ciprofloxacin, piperacillin-tazobactam and ceftazidime (Table 4).

**FIGURE 3:**
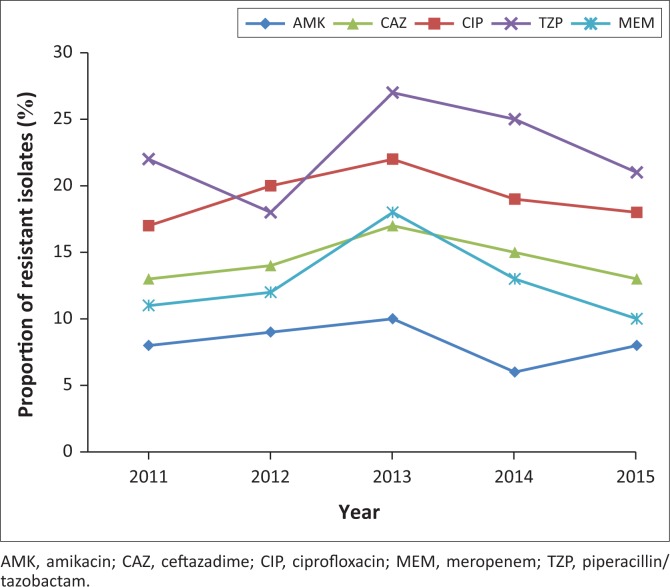
Resistance profile of *P. aeruginosa* (2011–2015).

Accumulative average resistance to piperacillin-tazobactam, ceftazadime, ciprofloxacin, meropenem, and amikacin observed in *P. aeruginosa* was 1706 (15.2%), 1601 (14.2%), 1501 (13.4%), 1090 (9.7%) and 716 (6.3%), (*p* < 0.001) (Table 4). Although 840 (7.5%) samples of *P. aeruginosa,* were resistant to colistin, this was not confirmed by reference or molecular methods. Over the five-year period, an increase in the total number of *P. aeruginosa* was noted (*p* = 0.002) (Figure 1). During 2013–2015, decreasing trends in resistance patterns were observed in *P. aeruginosa* to ceftazadime (from 17% to 13%, *p* = 0.004), piperacillin-tazobactam (from 27% to 21%, *p* < 0.001), meropenem (from 18% to 10%, *p* < 0.001), ciprofloxacin (from 22% to 18%, *p* = 0.002), and amikacin (from 10% to 8%, *p* < 0.001) (Figure 3).

During the five-year period, the average accumulative resistance rate to available carbapenems observed in *Enterobacter* spp. was 5%. Resistance to ciprofloxacin was greater in *E. cloacae* (16%) compared to *E. aerogenes* (8%) (data not shown).

**FIGURE 4:**
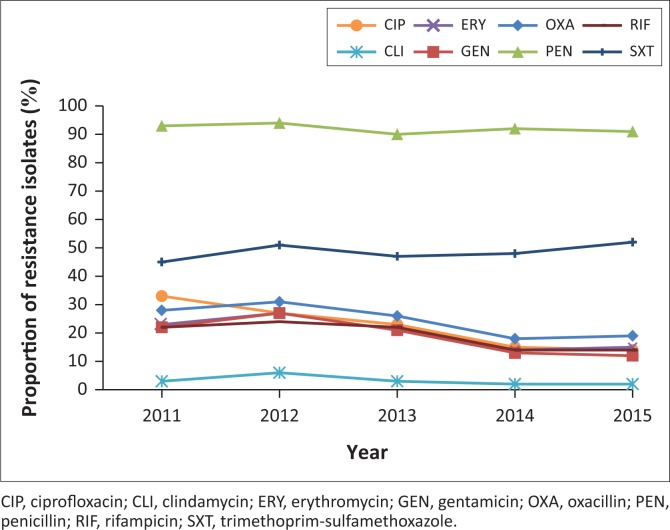
Resistance profile of *S. aureus* in the ESKAPE group (2011–2015).

As illustrated in Figure 4, rates of MRSA varied between 18% and 31% per year. Overall, a decreasing trend in the proportion of MRSA was observed between 2011 and 2014, 28% to 18% (*p* < 0.001). Decreasing resistance rates or trends across several antibiotic classes, namely gentamicin, clindamycin, ciprofloxacin, erythromycin and rifampicin, were also noted. No resistance to vancomycin, teicoplanin and linezolid was observed. Of the 24 495 isolates of *S. aureus* isolated over the study period, 22 596 (92%) were resistant to penicillin, 12 168 (49%) were resistant to co-trimoxazole, 6034 (24%) were resistant to cloxacillin, 5352 (21.8%) were resistant to ciprofloxacin, 4916 (20%) were resistant to erythromycin, 4702 (19%) were resistant to rifampicin, and 917 (3.7%) were resistant to clindamycin. All isolates of *E. faecium* (2217) were susceptible to vancomycin and linezolid.

**FIGURE 5:**
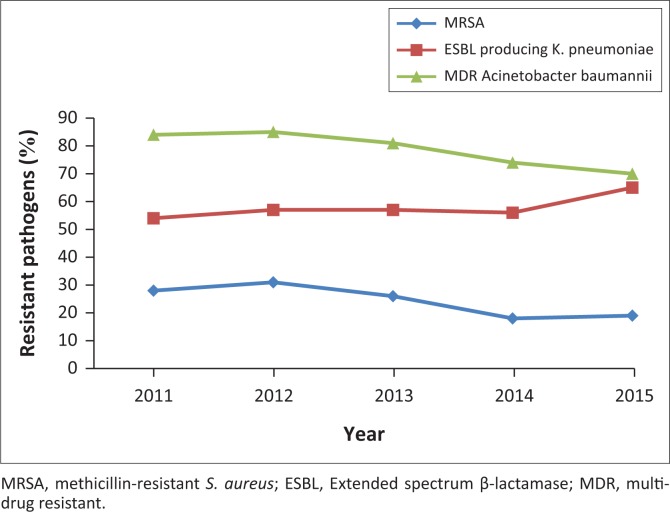
Distribution of highly resistant ESKAPE pathogens (2011–2015).

ESBL-producing *K. pneumoniae,* increased from 54.9 in 2011 to 65.5% in 2015 (*p* = 0.018). MRSA decreased from 31.4% in 2012 to 19.1% in 2015 (*p* < 0.001). MDR *A. baumanni* decreased from 85% in 2012 to 70% in 2015 (Figure 5). Overall, during the five-year period 6351 (79.2%) of all *A. baumannii* were MDR, 6034 (24.6%) of all *S. aureus* were MRSA and 8511 (59.5%) of *K. pneumoniae* were ESBL-producers.

## Discussion

Antimicrobial resistance is now a global pandemic that threatens the health, economic and social well-being of every individual.^13^ Antimicrobial resistance among bacterial pathogens is increasing globally at a rapid rate, including South Africa.^14^ Data from the Centers for Disease Control and Prevention indicate that the six ESKAPE bacteria are responsible for two-thirds of all healthcare-associated infections.^15^ The ESKAPE group of pathogens feature among the critical and high priority pathogens on the WHO global PPL. In this study, we report on five-year surveillance of ESKAPE pathogens and describe the incidence and resistance profiles of the ESKAPE pathogens in KwaZulu-Natal, South Africa.

Effective infection prevention and control bundles, comprising hand hygiene, contact precautions and a change in antibiotic prescription culture, advocated locally and internationally may have contributed to the possible decline in MRSA rate;^16^ however, the number of ESBL-producing *K. pneumoniae* increased. Infection prevention and control interventions should impact on decreasing both pathogens, but a notable increase in the number of ESBL-producing *K. pneumoniae* was observed. The use of third-generation cephalosporins, as part of the Integrated Management of Childhood Illness Guidelines^17^ and Sexually Transmitted Infections Management Guidelines in South Africa,^18^ may have contributed to the increase in ESBL-producing *K. pneumoniae*. Third-generation cephalosporins are administered empirically at the discretion of a community centre healthcare professional on suspicion of meningitis, sepsis or as part of syndromic management for sexually transmitted infections. In our study, the percentage resistance of 24% to methicillin observed in *S. aureus* was lower than the 46% that was described by Perovic et al., in 2015.^16^ MRSA proportions exceeded 20% in all WHO regions, and in some countries it exceeded 80%.^1^ The epidemiology of MRSA in Africa was highlighted in a systematic review of 263 articles. Prevalence of MRSA in Africa varied from 12% in Tunisia to 82% in Egypt.^16^ Our findings confirm that vancomycin resistance among *S. aureus* is yet to be encountered in KwaZulu-Natal. Decreasing trends in antimicrobial resistance to erythromycin, clindamycin, rifampicin and ciprofloxacin were observed in *S. aureus* during the study period. NHLS public sector susceptibility data (January–December 2009) presented by Crowther et al. showed varying rates of MRSA.^19^ The data obtained were from eight laboratories located in academic hospitals in South Africa, excluding KwaZulu-Natal. Rates of cloxacillin resistance in *S. aureus* varied between 24% and 84%. Additionally, clindamycin resistance reported in that study varied between 15% and 70% in comparison to less than 3% observed in our analysis.

*K. pneumoniae* was the most common Gram-negative organism isolated from the ESKAPE group and during the study period *K. pneumoniae* accounted for almost one-third of all ESKAPE organisms isolated from blood. In a 2015 global surveillance report by Healthcare-associated Infections Surveillance Network and United States National Healthcare Safety Network 8.7% and 9.9% of all hospital acquired infections were attributable to *K. pneumoniae*^20^. A cause for concern in this study is the steady increase in antimicrobial resistance to amoxicillin-clavulanate and third-generation cephalosporins reflecting inhibitor-resistance and ESBL production in *K. pneumoniae.* Perovic et al. reported similar proportions of ESBL-producing K. *pneumoniae* (68.3%) from bloodstream infections over a four-year period.^21^ In another South African study, Dramowski et al. reported that 75.7% and 78.3% of all *K. pneumoniae* isolated from paediatric community-acquired and hospital-acquired bloodstream infections were ESBL producers.^22^ According to a WHO report in 2014, resistance rates of *K. pneumoniae* to third-generation cephalosporins varied between 8% and 77% in Africa.^1^ Extended spectrum *β*-lactamase (ESBL) production in *Escherichia coli* and *K. pneumoniae* have been reported in Thailand (rate between 3.0% and 23.1%), China (65% and 31.9%), and India (67.0% and 55.0%).^23^ WHO reported that resistance to third-generation cephalosporins observed in *K. pneumoniae* was greater than 30% worldwide and greater than 60% in some countries.^1^

The rise in ciprofloxacin resistant *K. pneumoniae* in 2015 could be due to increased use of ciprofloxacin for treatment of ESBL-producing *K. pneumoniae* infections. More significantly, ciprofloxacin is used as a first-line therapy for uncomplicated urinary tract infections in the South African Standard Treatment Guidelines.^10^ During the period 2010–2012, national sentinel site surveillance for antimicrobial resistance in *K. pneumoniae* from South Africa was performed and 46.5% of all isolates were resistant to ciprofloxacin.^21^ Due to the large number of quinolone-resistant and ESBL-producing *K. pneumoniae*, treatment for severe *K. pneumoniae* infections rely on carbapenems.^24^ Perovic et al. noted less than 6% resistance rate to carbapenems in *K. pneumoniae* during 2010–2012.^21^ With reference to our study, although resistance to carbapenems was generally low between 2011 and 2014, a significant increase of 16% was noted in 2015, indicative of an emerging problem. Massive surveillance gaps in documenting carbapenem-resistant *K. pneumoniae* from Africa exist and this is a great challenge. In 2014, the WHO reported that 0% – 4% of *K. pneumoniae* from Africa were resistant to carbapenems; however, these data were obtained from only four countries^1^ on the African continent. Increasing carbapenem resistance in *K. pneumoniae* during 2015 could very well represent the global trends in increasing resistance of Enterobacteriacae to carbapenems. The WHO has highlighted alarming rates of carbapenem resistance in *K. pneumoniae*, exceeding 50% in some countries of the eastern Mediterranean and Europe.^1^ Limited therapeutic options to treat infections due to carbapenem-resistant *K. pneumoniae* are among the greatest challenges facing clinicians. Therapeutic options include colistin, but a recent Italian study showed that among 178 carbapenemase-producing *K. pneumoniae* isolates from different hospitals, 43% were also resistant to colistin.^25^ Tigecycline is not available for use in the public healthcare sector in KwaZulu-Natal; therefore, susceptibility and resistance data for this drug were not analysed in this study. The only remaining intervention to prevent dissemination of MDR organisms, such as carbapenemase-producing *K. pneumoniae*, is infection control.^24^

The significant decrease in resistance observed in *P. aeruginosa* to many of the anti-pseudomonal antimicrobial agents may be attributed to the fact that the majority of *P. aeruginosa* was isolated from non-sterile sites. It is well known that *P. aeruginosa* is a long-term coloniser and can persist for prolonged periods of time; hence, many clinicians opt not to administer systemic antimicrobials in a patient who is otherwise clinically well. The emergence and spread of antibiotic-resistant Gram-negative pathogens can be attributed to antibiotic selective pressure;^26^ therefore, the likelihood of developing MDR *P. aeruginosa* is reduced when no antimicrobials are administered for colonising bacteria.

Greater than 70% rates of resistance were observed in *A. baumanni* for most antimicrobials tested, except gentamicin, amikacin and colistin, where resistance was 68%, 18% and 4%, respectively. Susceptibility to amikacin and colistin remained stable for *A. baumannii* during the five-year period. The propensity for development of resistance to a drug is lower when it is used in combination than with monotherapy. This could explain the stable susceptibility to amikacin as it is only used as part of a dual therapy and never alone in South Africa. Colistin susceptibily could be due to its lack of availability or restricted use in the public health sector in South Africa. A motivation and prior approval is mandatory prior to drug administration; hence, most clinicians are unable to access the drug. Additionally, in the majority of patients with *A. baumannii*, treatment is not offered as most *Acinetobacter* species are considered long-term colonisers of low virulence potential and are intrinsically resistant to all but a few antimicrobial agents.^27,28^ Therapy for these colonisers would only lead to the emergence of other MDR organisms. Even in the face of directed therapy, *Acinetobacter* has the ability to develop resistance,^27^ and the isolation of colistin-resistant subpopulations of *Acinetobacter* is of great concern.^28^ Drawing a comparison to the global burden of MDR *A. baumannii*, a recent systemic review and meta-analysis by Bialvaei et al. (2017) highlighted that the pooled prevalence of MDR *A. baumannii* was 72% annually, with frequencies of between 22% and 100%,^29^ similar to the findings in the present study.

### Limitations

There are a number of limitations of this study. The total number of samples received by all the laboratories during the study period was not available. There were deficiencies in data collection for 2014 due to an upgrade of microbiological diagnostic systems, resulting in unrecoverable surveillance data. Additionally, although this surveillance reflects nine hospitals across two districts in KwaZulu-Natal, the private health sector was not included, and the results reflect only the public health sector in KwaZulu-Natal, South Africa.

### Conclusion

This is the first study describing resistance trends in WHO global priority pathogens within KwaZulu-Natal. The high prevalence rates of antimicrobial resistance observed in ESKAPE pathogens signal the need to improve antimicrobial stewardship and infection prevention and control programmes in the region.
